# Supplementary material for: Children of Smoking and Non-Smoking Households’ Perceptions of Physical Activity, Cardiorespiratory Fitness, and Exercise
Source: Children (Basel). 2021 Jun 26;8(7):552. doi: 10.3390/children8070552 (PMC8307148; doi:10.3390/children8070552)
Supplement: Supplementary file 1 [file children-08-00552-s001.zip › children-1224878-supplementary.pdf]

children MDPI

S1. Description and Rationale for Focus Group Questions and Activities in Relation to the Research Questions.

Icebreaker

Children were asked, 'Write down on the sticky note three words that you think describe physical activity.' What PA meant to the children was explored, before defining PA as 'Any body movement with our muscles that uses energy'.

RQ1. What are children from smoking and non-smoking households' reasons for being physically active?

To ascertain children's reasons for being physically active, children were asked about their favourite physical activities and to discuss what they enjoy and dislike about PA. In order to pose the research question in a child-accessible way, the following question was used: 'I'm going to ask you to have a think, then choose which physical activity you enjoy the most and tell me why.' Reasons for being active were explored further with prompts, such as 'What do you enjoy about the activity?'

RQ2. What are children from smoking and non-smoking households' attitudes towards physical activity, exercise, and fitness?

To elicit children's responses regarding their attitude towards physical activity, exercise, and fitness, interactive tasks and a range of questions were used in conjunction. A key step taken towards answering this research question was to establish the difference between PA, exercise, and fitness. Questions used to orientate the children with the key terms were used, for example 'What does fitness mean?', before the researcher defined the key terms in a child-appropriate way for clarity. A number of focus group questions mapped onto the second research question, including 'Is it important to be physically fit?', with prompts such as 'Is it important to you?'.

The 'show me' picture activity allowed children to show how a certain activity made them feel, using the PCERT scale [54] they had experienced in an earlier aspect of the overall PhD programme of study. Children were shown photographs of children participating in different physical activities (Figure S1), including playing on park apparatus, gymnastics, sprinting, walking, and swimming. The activities were selected to include a range of metabolic equivalent of tasks (METs) from casual walking, MET 3.6, to sprinting, MET 10 [148] but also to incorporate a range of components of fitness, including cardiorespiratory fitness, muscular strength, flexibility, agility, balance, power, speed, and coordination.

Children were asked to place the photographs on the scale to demonstrate how exerting they found each activity, with the following instruction: 'Look at this scale showing how hard an exercise is, with the easiest level of work at the bottom, and the hardest at the top, you might remember it from the fitness challenge. I'm going to give you some pictures of children doing various activities or sports and I'd like you to decide how hard you would find each activity. When you're ready, come and place the pictures on the scale, showing how hard you would find each activity.'

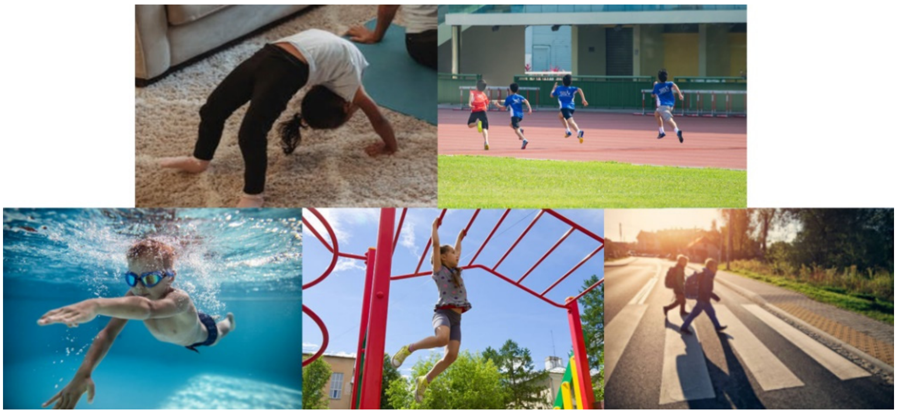

Figure S1. Pictures used in the exercise 'show me' task. Pictures demonstrate gymnastics (crab), running (sprinting), swimming, playing on park apparatus (monkey bars), walking (to/from school). Pictures (copyright free) are for illustrative purposes and are not the actual pictures used, as these could not be shared due to copyright.

RQ3. What are the barriers and facilitators to a child's ability to be physically active and does this differ for children from smoking and non-smoking homes?

Coloured post-it-notes and a flipchart were used as interactive resources. Children were asked to write down things that 'help' them to be physically active on the green post-it-note, and things that 'stop' them being physically active on the red post-it-note. This was followed up with a question about how adults can limit or facilitate children's physical activity.

RQ4. What are children's perceptions of their own fitness and physical ability and does this differ for children from smoking and non-smoking homes?

The fourth research question was answered with a mixture of open questions and interactive activities. The interactive activity using the PCERT and photographs, as described above, was also used to answer this research question. In addition, questions centred around fitness were used, such as 'How physically fit do you feel, on a scale of 1-10? With 1 being not very fit at all, 10 being the fittest you could be.'
